# USING COMMUNITY-BASED MODELS TO PROVIDE INTEGRATED CARE FOR RURAL OLDER ADULTS

**DOI:** 10.1093/geroni/igz038.2038

**Published:** 2019-11-08

**Authors:** Martha R Crowther, Cassandra D Ford

**Affiliations:** 1 The University of Alabama, Tuscaloosa, Alabama, United States; 2 The University of Alabama, Capstone College of Nursing, Tuscaloosa, Alabama, United States

## Abstract

Rural elders are one of the most at-risk populations for experiencing physical and mental health problems. In many rural communities, there are no psychosocial services available to meet the needs of the rural elderly. To provide rural older adults with integrated healthcare, we build upon our existing community-based infrastructure that has fostered community capacity for active engagement in clinical activities and has served as a catalyst to increase participation of rural older adults in clinical services. Our rural community model draws upon the role of culture in promoting health among rural older adults to provide rural service delivery. This model is built upon our network of partnerships with surrounding communities, including potential research participants, community-based organizations, community leaders, and community health-care systems and providers. By engaging the community we can create a sustainable system that will encourage rural older adults to utilize the health care system at a higher rate.

